# Supplementary material for: Assessment of in vitro activities of novel modified antimicrobial peptides against clarithromycin resistant Mycobacterium abscessus
Source: PLoS One. 2021 Nov 15;16(11):e0260003. doi: 10.1371/journal.pone.0260003 (PMC8592419; doi:10.1371/journal.pone.0260003)
Supplement: Attachment — Submitted filename: Response to reviewer_FINAL.docx [file pone.0260003.s003.docx]

**Response to reviewer comments**

Thank you very much for your comments and suggestions. We would like to answer your comments and suggestions as follows:

**Journal Requirements:**

*When submitting your revision, we need you to address these additional requirements.*

*1. Please ensure that your manuscript meets* PLOS ONE's style requirements*, including those for file naming. The PLOS ONE style templates can be found at*

[*https://journals.plos.org/plosone/s/file?id=wjVg/PLOSOne_formatting_sample_main_body.pdf*](https://journals.plos.org/plosone/s/file?id=wjVg/PLOSOne_formatting_sample_main_body.pdf) *and*

[*https://journals.plos.org/plosone/s/file?id=ba62/PLOSOne_formatting_sample_title_authors_affiliations.pdf*](https://journals.plos.org/plosone/s/file?id=ba62/PLOSOne_formatting_sample_title_authors_affiliations.pdf)

**Answer:** The manuscript formatting has been checked and the format revised accordingly. We hope that the revised version of the manuscript is according to PLOS ONE's style requirements.

*2. Please provide additional details regarding participant consent to collect blood samples for isolation of RBC and PBMCs. In the Methods section, please ensure that you have specified how consent was obtained or whether the research ethics committee or IRB specifically waived the need for their consent.*

**Answer:** A sentence has been added to the Materials and methods section to state this information, as follows:

(Lines 93-96) Informed consent was not required for this study. All specimens including isolates and blood samples were obtained from routine practice in which patient information was deidentified. The study protocol was approved by the Khon Kaen University Ethics Committee for Human Research (HE611496). The document in which the IRB specifically waived the need for their consent is contained in the supplemental file.

*3. We suggest you thoroughly copyedit your manuscript for language usage, spelling, and grammar. If you do not know anyone who can help you do this, you may wish to consider employing a professional scientific editing service.*

*Whilst you may use any professional scientific editing service of your choice, PLOS has partnered with both American Journal Experts (AJE) and Editage to provide discounted services to PLOS authors. Both organizations have experience helping authors meet PLOS guidelines and can provide language editing, translation, manuscript formatting, and figure formatting to ensure your manuscript meets our submission guidelines. To take advantage of our partnership with AJE, visit the AJE website (*[*http://learn.aje.com/plos/*](http://learn.aje.com/plos/)*) for a 15% discount off AJE services. To take advantage of our partnership with Editage, visit the Editage website (*[*www.editage.com*](http://www.editage.com/)*) and enter referral code PLOSEDIT for a 15% discount off Editage services.  If the PLOS editorial team finds any language issues in text that either AJE or Editage has edited, the service provider will re-edit the text for free.*

*Upon resubmission, please provide the following:*

*The name of the colleague or the details of the professional service that edited your manuscript*

*A copy of your manuscript showing your changes by either highlighting them or using track changes (uploaded as a *supporting information* file)*

*A clean copy of the edited manuscript (uploaded as the new *manuscript* file)*

**Answer:** English usage of the final version of this manuscript was reviewed by Emeritus Professor James A Will, University of Wisconsin-Madison for editing the MS via Publication Clinic KKU, Thailand (jawenator@gmail.com). He is the senior editor for the Faculty of Medicine and successfully edits 80-100 medical manuscripts for this Faculty a year. The manuscript has further been revised for typos and grammatical errors. We hope this new version meets the high standards of PLOS ONE.

*4. Thank you for stating the following financial disclosure:*

*“The funders had no role in study design, data collection and analysis, decision to publish, or preparation of the manuscript.”*

*At this time, please address the following queries:*

*a) Please clarify the sources of funding (financial or material support) for your study. List the grants or organizations that supported your study, including funding received from your institution.*

*b) State what role the funders took in the study. If the funders had no role in your study, please state: “The funders had no role in study design, data collection and analysis, decision to publish, or preparation of the manuscript.”*

*c) If any authors received a salary from any of your funders, please state which authors and which funders.*

*d) If you did not receive any funding for this study, please state: “The authors received no specific funding for this work.”*

*Please include your amended statements within your cover letter; we will change the online submission form on your behalf.*

**Answer:** We added the funding to the on online submission form, as follows:

This study was supported by the Invitation Research, Faculty of Medicine, Khon Kaen University (Grant number: IN62307) and National Research Council of Thailand (Grant number: NRC MHESI 483/2563). The funders had no role in study design, data collection and analysis, decision to publish, or preparation of the manuscript.

*5. Thank you for stating the following in the Funding Section of your manuscript:*

*“This study was supported by the Invitation Research, Faculty of Medicine, Khon Kaen University (Grant number: IN62307) and Research and Diagnostic Center for Emerging Infectious Diseases (RCEID), Khon Kaen University, Khon Kaen, Thailand.”*

*We note that you have provided additional information within the Funding Section that is not currently declared in your Funding Statement. Please note that funding information should not appear in the Acknowledgments section or other areas of your manuscript. We will only publish funding information present in the Funding Statement section of the online submission form.*

*Please remove any funding-related text from the manuscript and let us know how you would like to update your Funding Statement. Currently, your Funding Statement reads as follows:*

*“The funders had no role in study design, data collection and analysis, decision to publish, or preparation of the manuscript.”*

*Please include your amended statements within your cover letter; we will change the online submission form on your behalf.*

**Answer:** We removed funding-related text and the information of competing interests from the manuscript and these sentences are stated in the online submission form.

**Reviewer #1:**

**Q#1** *M abscessus does exists as biofilms during infections and the resistance pattern of biofilm vs broth grown Mab are highly varied. Why have the authors restricted to the broth form of MAB.*

**Answer:** The broth state of the experiment is the easiest way to control environmental condition of the experiment and drug exposure to the pathogens. The point that the experimental condition does not cover the biofilm producing state is the limitation of our study. We have added this limitation in the last paragraph of the Discussion section as follows:

(Lines 408-415) *Mab* is a prolonged intracellular pathogen with varied ability to produce biofilm, biofilm-forming smooth morphotypes and non-biofilm forming rough morphotypes [63]. Although various *Mab* isolates of both rough and smooth morphotypes were included in these experiments, DST were not determined in the biofilm producing state of *Mab*. These experiments were only under *in vitro* conditions; the *in vivo* response of AMPs and CLA could be varied depending on the host environment. Additional studies that investigate the in-depth assessment with a larger number of isolates, including the biofilm producing state and *in vivo* experiments are likely warranted.

**Q#2** *Line 312 talks about anti-biofilm effect of AMPs on Streptococcus. The in vitro synergism and anti-microbial effect may be very different from the clinical scenario unless screened on a biofilm model and warrants further investigation before moving forward. Discussion needs to throw light on specific aspects like treatment response and duration due to use of AMP.*

**Answer:** *Mab* is a prolonged intracellular pathogen with varied ability to produce biofilm, biofilm-forming smooth morphotypes and non-biofilm forming rough morphotypes [63]. Although various *Mab* isolates of both rough and smooth morphotypes were included in these experiments, DST were not determined in the biofilm producing state of *Mab*. This is the limitation of our study. We stated this as the limitation in the discussion (Lines 408-411).

**Q#3** Please highlight other drug resistance if any identified through sequencing and any similarities and differences in mutation patterns among cluster of inducible or acquired resistant strains.

**Answer:** The isolates that we included are the CLA-resistant *Mab* isolates. The susceptibility test for amikacin which is another important drug for treatment of *Mab* infection is also available. We have included the susceptibility results of the additional drug in the Table 2. The drug susceptibility test based on WGS analysis is still pending as the mutation database is not compete and the analysis would take a very long time to finish. We have thus separated this objective out of the scope of this study.

**Q#4** Line 51 – Mention the duration of treatment

**Answer:** A new text has been added to the Introduction section to state, as follows:

(Lines 52-53) The duration of treatment for *Mab* infection depends on the clinical syndrome and lasts from 4 weeks to 12 months.

**Q#5** Line 58 – Please mention which other bacteria and if any mycobacterial species were tested with AMPs

**Answer:** In this study, no other *Mycobacterium* species was tested but references are provided in our text. A new text has been added to the Introduction section to state and the references have been added, as follows:

(Lines 61-64) Several research teams have reported AMPs activity against *Mycobacterium tuberculosis* [10-27] and other NTMs such as *Mycobacterium avium* [12, 19, 28, 29], *Mycobacterium smegmatis* [12, 20, 30], *Mycobacterium vaccae* [31], *Mycobacterium bovis* [32] and *Mycobacterium marinum* [33].

**Q#6** Line 62 – “Polydim-I reduced 40 to 50% of Mab subsp. massiliense infected macrophage”. This sentence refers to mycobacteria or macrophages. It is confusing.

**Answer:** This sentence refers to macrophages. We have modified this sentence as follows:

(Lines 67-69) Polydim-I treatment of macrophages infected with different *Mab* subsp. *massiliense* strains reduced the bacterial load by 40 to 50% [35].

**Q#7** Table2 shows MAB13 as acquired while it is increasing from day 3 to day 14 with respect to clarithromycin.

**Answer:** Inducible resistance was inferred by changes in MIC values from “susceptible” at day 3 to “resistant” at day 14. Isolates that were resistant on day 3 and thereafter were regarded as demonstrating acquired resistance. Therefore, MAB13 had resistance from day 3 to day 14, which was defined as acquired resistance.

**Q#8** Several typos and grammar improvement for discussion recommended – line 373 (regarding); 378 – patients and administration etc.,

**Answer:** English usage of the final version of this manuscript will be reviewed by Emeritus Professor James A Will, University of Wisconsin-Madison for editing the MS via Publication Clinic KKU, Thailand (jawenator@gmail.com). The current revised version of the manuscript has further been revised for typos and grammatical errors. We hope this new version meets the high standards of PLOS ONE.
