# Supplementary material for: Assessment of in vitro activities of novel modified antimicrobial peptides against clarithromycin resistant Mycobacterium abscessus
Source: PLoS One. 2021 Nov 15;16(11):e0260003. doi: 10.1371/journal.pone.0260003 (PMC8592419; doi:10.1371/journal.pone.0260003)
Supplement: Attachment — Submitted filename: Response to reviewer_Final.docx [file pone.0260003.s004.docx]

Response to reviewer comments

Thank you very much for your comments and suggestions. We would like to answer your comments and suggestions as follows:

**Journal Requirements:**

Please review your reference list to ensure that it is complete and correct. If you have cited papers that have been retracted, please include the rationale for doing so in the manuscript text, or remove these references and replace them with relevant current references. Any changes to the reference list should be mentioned in the rebuttal letter that accompanies your revised manuscript. If you need to cite a retracted article, indicate the article’s retracted status in the References list and also include a citation and full reference for the retraction notice.

**Answer:** Thank you very much for your suggestions. We checked the references have been corrected and completed. The reference list has been rearranged because two references have been added according to the reviewer suggested as follows:

(Lines 450-455)

6.	Strnad L, Winthrop KL. Treatment of *Mycobacterium abscessus* Complex. Semin Respir Crit Care Med. 2018;39(3):362-76. https://doi.org/10.1055/s-0038-1651494 PMID: 30071551

7.	Weng YW, Huang CK, Sy CL, Wu KS, Tsai HC, Lee SS. Treatment for *Mycobacterium abscessus* complex-lung disease. J Formos Med Assoc. 2020;119 Suppl 1:S58-S66. https://doi.org/10.1016/j.jfma.2020.05.028 PMID: 32527504

**Comments to the Author**

Review Comments to the Author

Please use the space provided to explain your answers to the questions above. You may also include additional comments for the author, including concerns about dual publication, research ethics, or publication ethics. (Please upload your review as an attachment if it exceeds 20,000 characters)

Reviewer #1:

1. Please add "The drug susceptibility test based on WGS analysis is still pending as the mutation database is not compete and the analysis take very long time to finish. Then we have separated this objective out of the scope of this study" to the main text.

**Answer:** We have added these sentences as the limitation in the Discussion section as follows:

“The drug susceptibility test based on WGS analysis is still pending as the mutation database is not completed and the analysis take very long time to finish. Then, we have separated this objective out of the scope of this study.” (Lines 405-407).

2. Please add reference to "The duration of treatment for Mab infection depends on the clinical syndrome and lasts from 4 weeks to 12 months" in line 51

**Answer:** The reference has been added, as follows:

The duration of treatment for *Mab* infection depends on the clinical syndrome and lasts from 4 weeks to 12 months [6, 7]. (Lines 52-53)

6.	Strnad L, Winthrop KL. Treatment of *Mycobacterium abscessus* Complex. Semin Respir Crit Care Med. 2018;39(3):362-76. https://doi.org/10.1055/s-0038-1651494 PMID: 30071551

7.	Weng YW, Huang CK, Sy CL, Wu KS, Tsai HC, Lee SS. Treatment for *Mycobacterium abscessus* complex-lung disease. J Formos Med Assoc. 2020;119 Suppl 1:S58-S66. https://doi.org/10.1016/j.jfma.2020.05.028 PMID: 32527504

3. Spell check to be rigorously done

**Answer:** Thank you very much for your suggestions. Before submission, the final version of this revised manuscript was reviewed by Emeritus Professor James A Will, University of Wisconsin-Madison for editing the MS via Publication Clinic KKU, Thailand. The spelling check have been done. All typographical errors have been corrected.
